# An analysis of expression patterns of genes encoding proteins with catalytic activities

**DOI:** 10.1186/1471-2164-8-232

**Published:** 2007-07-12

**Authors:** Murat Cankaya, Ana Martinez Hernandez, Mehmet Ciftci, Sukru Beydemir, Hasan Ozdemir, Harun Budak, Ilhami Gulcin, Veysel Comakli, Tufan Emircupani, Deniz Ekinci, Muslum Kuzu, Qiuhong Jiang, Gregor Eichele, Omer Irfan Kufrevioglu

**Affiliations:** 1 Genes and Behavior Department, Max Planck Institute of Biophysical Chemistry, Am Fassberg 11, 37077 Goettingen, Germany; 2 Department of Chemistry, Faculty of Arts and Science, Atatürk University, 25240 Erzurum, Turkey

## Abstract

**Background:**

*In situ *hybridization (ISH) is a powerful method for visualizing gene expression patterns at the organismal level with cellular resolution. When automated, it is capable of determining the expression of a large number of genes.

**Results:**

The expression patterns of 662 genes that encode enzymes were determined by ISH in the mid-gestation mouse embryo, a stage that models the complexity of the adult organism. Forty-five percent of transcripts encoding metabolic enzymes (n = 297) showed a regional expression pattern. A similar percentage was found for the 190 kinases that were also analyzed. Many mRNAs encoding glycolytic and TCA cycle enzymes exhibited a characteristic expression pattern. The annotated expression patterns were deposited on the Genepaint database and are retrievable by user-defined queries including gene name and sites of expression.

**Conclusion:**

The 662 expression patterns discussed here comprised gene products with activities associated with catalysis. Preliminary analysis of these data revealed that a significant number of genes encoding housekeeping functions such as biosynthesis and catabolism were expressed regionally, so they could be used as tissue-specific gene markers. We found no difference in tissue specificity between mRNAs encoding housekeeping functions and those encoding components of signal transduction pathways, as exemplified by the kinases.

## Background

When and where genes are expressed provides useful information about their function. This view forms the basis of several genome-scale projects that attempt the systematic localization of mRNAs and/or proteins expressed in tissues and cells [1,2]. When the products of genes remain within the cells that transcribe and translate them, localizing mRNAs is especially informative. Cell-autonomously acting proteins include transcription factors, transmembrane proteins and also the majority of metabolic enzymes. Although the expression patterns of many genes that encode enzymes have been determined on a gene-by-gene basis, the systematic effort used in the present study stands out because it not only provides a significant coverage of enzymes encoded in the genome but is conducted in a standardized fashion. In addition, expression sites are annotated and images are made accessible on a public website [3,4].

The biochemistry of glycolytic and TCA cycle enzymes has been extensively studied, but the expression patterns of the genes encoding these enzymes in a multicellular organism have not yet been systematically examined. Genes encoding glycolytic and TCA cycle enzymes are a particularly attractive target for a systematic analysis of expression patterns because their products form pathways. The glycolytic pathway is located in the cytosol and consists of a sequence of ten steps catalyzed by 22 different gene products, some of which are isozymes. The product of glycolysis (pyruvate) serves as starting material for the mitochondrial TCA cycle. The TCA cycle comprises eight distinct steps involving 20 gene products, several of which form multisubunit complexes.

While glycolysis and the TCA cycle illustrate pathways characterized by sequences of catalytic reactions, mammalian carbonic anhydrases (CAs) exemplify a single protein family, all the members of which catalyze the interconversion of carbon dioxide and bicarbonate [5]. Notably, 13 of the 16 CAs are catalytically active while the remaining three are CA-related polypeptides (CA-RP). These have high sequence homology to CAs but seem to lack enzymatic activity [6,7]. The three CA-RPs (CA VIII, X and XI) owe their putative lack of CA activity to substitutions of one or more active site residues [6,7]. CAs are located in different subcellular compartments: CA I, II, III, VII [5,8] and XIII are cytosolic enzymes [9]. CA IV [10] and XV [11] are anchored to the plasma membrane, while CA IX, XII [12] and XIV [13] are transmembrane proteins. Both CA VA and CA VB encode mitochondrial enzymes [14,15], while CA VI is a secreted protein [16,17]. CAs participate in a variety of physiological processes including pH regulation, CO2 and HCO3^- ^transport, ion transport, water and electrolyte balance, ureagenesis, gluconeogenesis and lipogenesis (reviewed in [5,18]).

We previously developed methods for determining the spatial expression patterns of genes by *in situ *hybridization (ISH) on a transcriptome-wide scale ([3,19]; see also[4] and [20]). Here we report the expression patterns of 662 genes that encode either catalytic or regulatory subunits of enzymes, representing a quarter of the 2700 gene products with catalysis-associated activity. Gene expression was analyzed on serially sectioned, stage E14.5 mouse embryos. The mid-gestation embryo provides an overview of the entire organism showing well-differentiated organs and tissues resembling those of the adult in their basic design. One can readily capture all organs in a set of 24 serial sections through such an embryo. The genetic programs underlying e.g. growth, terminal differentiation, cell migration and apoptosis are still developing at E14.5, so it is possible to study a wide range of biological processes [21].

This report has three aims. It provides a listing and a first-pass analysis of the expression of all 662 genes; it compares the expression patterns of glycolytic and TCA cycle genes; and it compares the expression patterns of 15 of the 16 known CAs in order to illustrate the astonishing diversity of expression patterns exhibited by members of a single multigene family.

## Results and discussion

### Expression patterns of mouse genes encoding proteins with catalytic activity

Under its "Molecular Function" category, Gene Ontology lists approximately 2700 gene products that exhibit activity associated with catalysis [22]. We have determined the expression patterns of 662 of the 2700 mRNAs in E14.5 mouse embryos using robotic ISH. The expression pattern of each gene was based on the analysis of ~ 24 sagittal sections, 25 μm thick and 150 μm apart. The products of the 662 genes included transferases, oxidoreductases, ligases and other enzymes (Table 1). Genes were selected on the basis of three not mutually exclusive criteria: (1) the genes scored as 'present' in a duplicate Affymetrix microarray chip (MA) hybridized with mRNA from E14.5 mouse embryos (MA data were deposited at NCBI GEO under accession number GSE6081); (2) all gene products encoding glycolytic and TCA cycle enzymes were selected; (3) for kinases, the intensity of the MA signal was ranked by the raw numerical value, and the 190 most strongly-expressed transcripts were selected.

**Table 1:** Classification of genes analyzed by type of catalytic activity

**GO term name (subcategory level 2)**	**Total number of GO gene products**	**Number of GO gene product represented in ISH data set**	**Percentage of GO covered by ISH data set**
Hydrolase activity	1011	188	18%
Transferase activity	908	261	28%
Oxidoreductase activity	433	114	26%
Ligase activity	146	62	42%
Lyase activity	72	56	77%
Helicase activity	59	4	6%
Isomerase activity	52	18	34%
Peroxidase activity	27	10	37%
RNA editase activity	4	3	75%
Genes with inferred activity		3	

All but three of the 662 genes analyzed can be classified into a particular Gene Ontology (GO) subcategory of enzymatic activity (Table 1). On the basis of their domain structures, Dip2a, Echdc1 and Echdc2 have inferred catalytic activities. The ISH data set used here represents between 18 and 77% of the GO subcategories. Also, one third of the murine kinome was represented by 190 kinases, a subcategory of transferases [23].

Of the 662 genes, 594 (90%) were expressed in one or more embryonic tissues (Table 2), while only 68 (10%) were not detected. One hundred and six (16%) were expressed ubiquitously (U); 297 (45%) were expressed regionally (R). One hundred and ninety-two (29%) transcripts fell into the "ubiquitous with pattern" (UWP) category (defined as an expression pattern in which transcripts are detected in most tissues and cells but expression is elevated in subregions within tissues). A similar distribution was observed when kinases only were scored; 45% of the kinases were regionally expressed and ~ 30% showed UWP expression. Thus, metabolic enzymes, often viewed as housekeeping proteins that should be expressed ubiquitously, and kinases, involved in signal transduction and hence expected to exhibit tissue-specific expression, showed similar degrees of regionalization of expression. Additional file 1 lists all 662 genes analyzed and provides information about their overall expression type using the nomenclature of Table 2 and complemented by a numerical score for expression level (not detected, or levels 1 [weak], 2 [medium] and 3 [strong]).

**Table 2:** Frequency of gene expression patterns

**ISH expression pattern**	**Number of cases**
Ubiquitous (U)	106
Regional (R)	297
Ubiquitous with pattern (UWP)	191
Not detected (N)	68

All ISH data generated for this study are fully accessible at the Genepaint database [4]. Data can be retrieved using gene name, gene symbol (provided in Additional file 1), accession number, Entrez Gene ID or Genepaint set ID (a specific accession number, unique to each data set deposited in the Genepaint database) [3,4]. Many of the expression patterns of the 662 genes are annotated in approximately 100 different tissues. As a result, patterns can be queried by site of expression. For example, genes expressed in liver and heart can be identified using the structure selection tool of the Genepaint database [4]. Images of individual sections of each set retrieved can be inspected using image viewers that are integrated into the database. For additional instructions on how to access Genepaint data see references [3,4,19].

**Figure 1:**
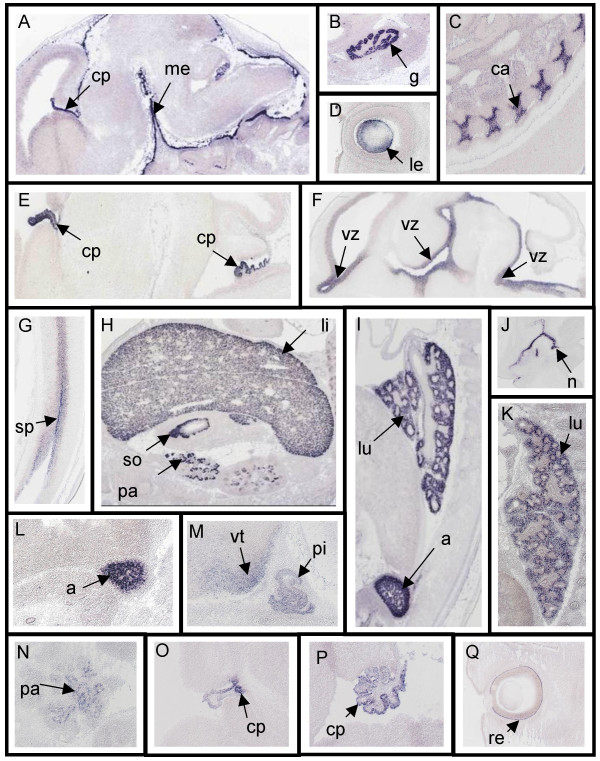
**Examples of tissue-specific molecular markers in the E14.5 mouse embryo**. **A **and **B**. *Prostaglandin D2 synthase (brain) *(*Ptgds*) (Genepaint set ID HD33, image 2C) expression is exclusive to the meninges (me), choroid plexi (cp, A) and gonads (g, B). **C. ***Lysyl oxidase-like 3 *(*Lox13*) (MH1813, 4A) is exclusively expressed in cartilage (ca). **D **and **E**. *Ribonuclease T2 (Rnaset2*) (MH1633, 1A, 5A) is a suitable marker for lens (le, D) and choroid plexi (cp, E). **F **and **G**. *Acetyl-Coenzyme A synthetase 2 (AMP forming)-like *(*Acas2l*) (MH1439, 4C, 4B) marks the ventricular zones of the nervous system (vz, F), including that of the spinal cord (sp, G), and liver (not shown). **H**. The gene *Carboxypeptidase N polypeptide 1 *(*Cpn1*) (MH1654, 5B) marks liver (li), pancreas (pa) and stomach (so). **I **and **J**. Expression of *Alcohol dehydrogenase 1 (class I) *(*Adh1*) (MH1744, 3D) is seen in adrenal gland (a, I), lung mesenchyme (lu, I) and nasal epithelium (n, J). **K **and **L**. *Phospholipase A2 *(*Pla2g2f*) (MH1625, 3A) expression is restricted to the lung (lu, K) and the adrenal gland (a, L). **M **and **N**. *Glucokinase *(*Gck*) (MH1608, 4C) is exclusive to the ventromedial nucleus of the hypothalamus (vt), preoptic area, anterior pituitary (pi, M) and pancreas (pa, N), **O – Q**. Expression of *Carbonic anhydrase 14 *(*Car14*) (MH1292, 3C, 1A) in choroids plexi (cp, O and P) and retina (re, Q).

Through our detailed analysis of the expression patterns, we identified numerous genes with tissue-specific expression, which can be used as molecular markers at E14.5 (Fig. 1A–Q). Lysyl oxidase-like 3 (Loxl3, Fig. 1C) is expressed in cartilage. This is consistent with earlier reports that implicated this enzyme in the biogenesis of connective tissues [24,25]. Acetyl-Coenzyme A synthetase 2 (AMP forming)-like (Acas2l) is strongly expressed in the ventricular zone of the developing nervous system (Fig. 1F, G) and in liver (Genepaint set ID MH1439, section 4C). This enzyme ligates acetate to coenzyme A to produce acetyl-CoA, a molecule essential in various metabolic pathways. Accordingly, Acas2l is reported to be strongly expressed in metabolically active tissues in adult mice [26]. Interestingly, however, it is not seen in adult liver [26]; apart from the CNS, liver is the only site at which Acas2l is expressed in the embryo. Expression in the strongly proliferating CNS ventricular zone may reflect the energy requirements of dividing cells. Carboxypeptidase N polypeptide 1 (Cpn1, Fig. 1H) exclusively marks the digestive system with strong expression in liver, intestine, pancreas and stomach. A previous study reports Cpn1 expression in embryos as early as E8.5 [27]. The ISH data in [25] also show expression at E10.5 and E13.5 in erythroid progenitor cells in the embryonic liver and at E16.5 in hepatocytes.

The subsequent sections of this report focus on glycolysis and TCA cycle genes. The salient features of the expression pattern are briefly described for each gene and, where warranted, patterns are related to those of other genes in the same pathway. Individual images of expression patterns are not provided in this paper since there are more than 500 images for the TCA cycle genes alone. Instead, the section below provides the Genepaint set ID and the section number leading to the correct image on the Genepaint database [4]. Genepaint set IDs (e.g. MH2297) can be entered in the search field of the homepage [4]. Clicking on the "go" button produces the "results" page on which the "set viewer" link is available. The appropriate section link (e.g. section 3A) can be selected in the image directory, generating a thumbnail of the section. This thumbnail can be magnified by clicking any one of three viewer buttons labelled "HTML", "Applet" or "Plugin".

### Expression patterns of glycolytic pathway enzymes

**Figure 2:**
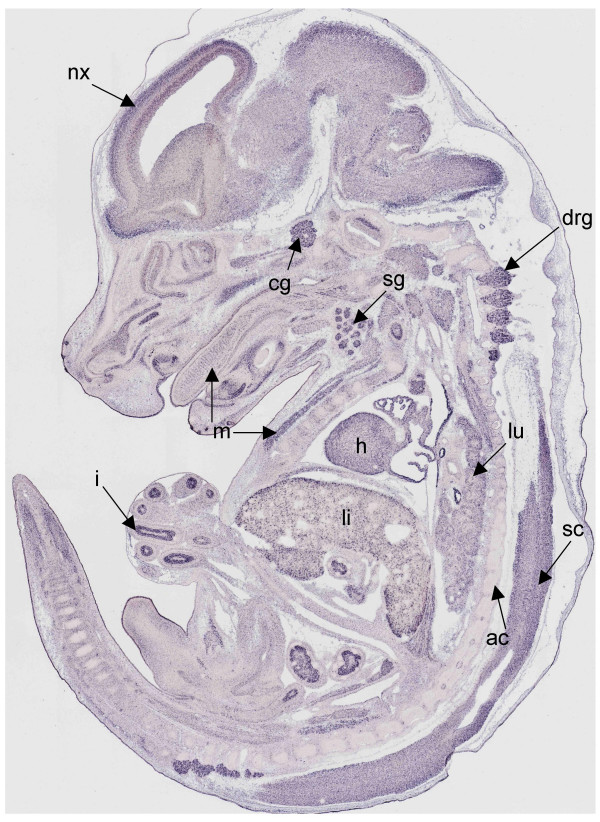
**Expression pattern of *Isocitrate dehydrogenase 3 (NAD+) alpha, (Idh3a)*, a representative example of a gene with a stereotypical energy metabolism pattern (SEMP)**. *Idh3a *(MH 965, 4C) is expressed widely in CNS, PNS, muscle, liver, kidney, lung, heart, pancreas, intestine, thymus, epithelia covering the alimentary and respiratory tract and whisker follicles. Relevant to the definition of SEMP is a relative regional elevation of expression, e.g. in the neocortex (nx), cranial ganglia (cg), dorsal root ganglia (drg), spinal cord (sc), heart (h), liver (li), lung (lu), intestine (i), salivary glands (sg) and muscles (m). Furthermore, a feature of a SEMP is the absence of expression in cartilage of the axial (ac) and appendicular skeletal anlage (not shown).

**Figure 3:**
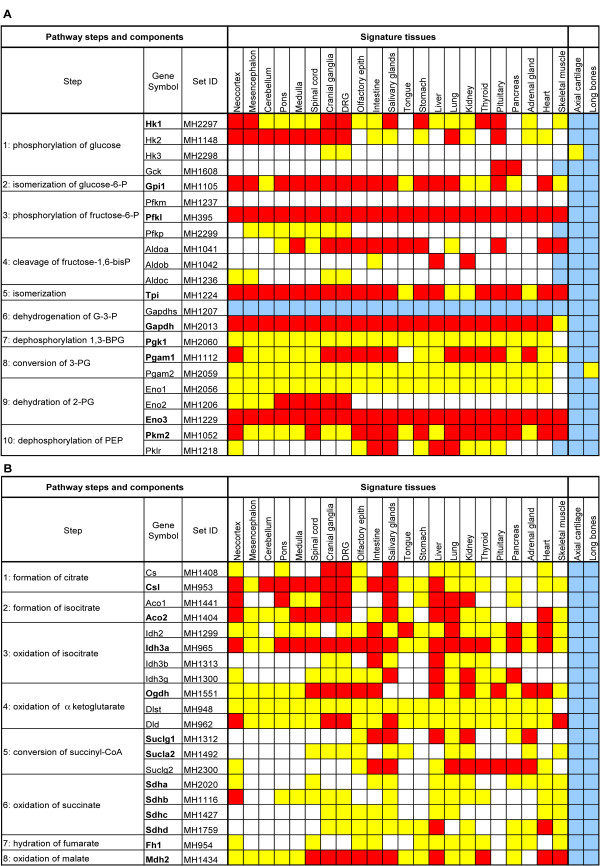
**Matrices representing the expression pattern of glycolysis (A) and TCA cycle (B) genes in signature tissues**. The expression level for each gene is shown in color. Red signifies strong expression; yellow, medium; and blank, weak expression. In cases where transcripts could not be detected, boxes are shown in blue. The Genepaint set IDs point to the data sets from which quantitative assessments were made. Genes showing a SEMP are in bold.

We found that many of the glycolytic and TCA cycle genes exhibited a recurrent pattern of expression, which we denoted the "Stereotypical Energy Metabolism Pattern" (SEMP). The SEMP, an example of which is shown in Fig. 2, is characterized by the following features. First, expression is seen in a set of 22 "signature tissues" (listed in the header of matrices A and B in Fig. 3) and expression strength is usually not constant across the signature tissues but varies among them. Second, expression is not detected in axial cartilage and long bones. The matrix shown in Fig. 3A is a schematic diagram of the expression of all glycolytic pathway components assessed in signature tissues, axial and limb cartilage. It can be seen that with the exception of aldolases (see below), at least one gene exhibits a SEMP (highlighted in bold in the Gene Symbol column of Fig. 3A) for each step in glycolysis. 9 of the 22 genes involved in glycolysis show a SEMP.

#### *Hexokinase 1, Hk1* (Genepaint set ID MH2297, section 3A)

This gene is broadly expressed throughout the embryo but signal strength is elevated in the superior and inferior colliculi, spinal chord, dorsal root- and cranial ganglia, and in the telencephalon.

#### *Hexokinase 2, Hk2* (MH1148, 3A)

The expression pattern in the nervous system strongly resembles that of *Hk1*, but in contrast to *hexokinase 1, Hk2 *is only weakly expressed outside the nervous system except in the epithelial component of the lung.

#### *Hexokinase 3, Hk3* (MH 2298, 3A)

Low and ubiquitous expression characterizes this gene, with some notable exceptions such as the cranial- and dorsal root ganglia (3D), which exhibit strong expression in scattered neurons. Expression is markedly elevated in the perichondrium of the axial skeleton (3B).

#### *Hexokinase 4, Gck* (MH1608)

Expression is observed in the anterior pituitary, in the ventromedial nucleus of the hypothalamus and in the preoptic area (4C). It is also seen the locus coeruleus (3A), the major site of noradrenalin production in the mammalian brain. Outside the CNS, Gck mRNA is seen in the primordium of the pancreas (4C). Among the four genes encoding the allosterically regulated hexokinases, *Gck *stands out because of its spatially restricted expression pattern.

#### *Glucose phosphate isomerase 1, Gpi1* (MH1105, 3A)

This gene is expressed in almost every part of the embryo and very strongly in some tissues. *Gpi1 *expression is particularly strong in the ventricular zone of the developing neocortex and spinal cord, in the motor column along the spinal cord, in dorsal root and cranial ganglia and in the cranial nerve nuclei. The retina (1B), olfactory epithelium, heart, muscles, liver and whisker follicles express *Gpi1*. Like *Gck*, *Gpi1 *is expressed in the locus coeruleus and in many midbrain neurons (2C).

#### *Phosphofructokinase muscle, Pfkm* (MH1237, 2D and 4B)

Generally, this gene is weakly expressed in many regions of the nervous system. The most striking expression is in skeletal muscles such as those of the hip (5C) and handplate (6A).

#### *Phosphofructokinase liver, Pfkl* (MH395, 3A)

This gene is strongly expressed. Transcripts are detected in liver, muscles, nervous system and the skeletal anlage. *Pfkl *transcripts are very abundant in the marginal zone of the cortex, where they form a patch-like pattern (5C). Other obvious sites of expression include midbrain, medulla, spinal cord, dorsal root- and cranial ganglia, thalamus and hypothalamus. Thymus, pancreas, kidney, adrenal gland and lung also express *Pfkl*.

#### *Phosphofructokinase platelet, Pfkp* (MH2299, 3A)

The defining feature of *Pfkp *expression is its restriction to the central and peripheral nervous systems. In the telencephalon, highly regional expression is seen in the piriform cortex (2A) and in scattered neurons of the vetromedial nucleus of the hypothalamus (4B). In thalamus, midbrain, pons and medulla, numerous single neurons express this gene with very prominent expression in the cranial nerve nuclei (e.g. 3A). Dorsal root ganglia, motor neurons in the spinal cord, retina and olfactory bulb also express *Pfkp*.

#### *Aldolase 1, A isoform, Aldoa* (MH1041, 4A)

*Aldoa *shows strong expression in all muscles, the cardiovascular system, kidney, and the axial and appendicular skeletal elements. In liver, numerous scattered cells also express *Aldoa *strongly. Transcripts are seen in ganglia, in the motor-column of the spinal cord and in the brain, where elevated expression is detected in the piriform cortex, pons and cranial nerve nuclei.

#### *Aldolase 2 B isoform, Aldob* (MH1042, 5B)

*Aldob*'s expression is highly regional; transcripts are found only in liver and the kidney tubules. There may be weak *Aldob *expression in the midgut (4A).

#### *Aldolase 3 C isoform, zebrin II, Aldoc* (MH1236, 4A)

Expression of this isoform is strong in the ventricular zone of neocortex (5B), hippocampus and spinal cord (4B). Strong expression is also seen in neuroepithelium lining the floor of the aqueduct and of the fourth ventricle (5A). In the case of the fourth ventricle, cells exiting the neuroepithelium express *Aldoc *(5A). Weaker *Aldoc *is seen in cranial and dorsal root ganglia as well as throughout the spinal cord. Notably, *Aldolase a*, *b *and *c *collectively constitute a SEMP when superimposed but none of them alone is a SEMP.

#### *Triosephosphateisomerase, Tpi* (MH1224, 4D)

*Tpi *is expressed throughout the embryo but signal strength varies. It is elevated in the ventricular zone of the cortex, in the pons, medulla, midbrain, lens, liver, heart, cranial ganglia and choroid plexi. A particular feature of the expression pattern is the scattered *Tpi*-positive cells located at the striatal-cortical interface (2A). In addition, this gene is expressed in the spinal cord, most strongly in the motor column (4C). *Tpi *transcripts are also detected in muscles, the PNS, piriform cortex, retina, epidermis, cerebellum, pituitary, lung, facial epithelium, intervertebral discs and joints of limbs.

#### *Glyceraldehyde 3-phosphate dehydrogenase spermatogenic, Gapdhs* (MH1207, 4B)

No expression of *Gapdhs *is detected at E14.5.

#### *Glyceraldehyde 3-phosphate dehydrogenase, Gapdh* (MH2013, 4C)

The expression pattern of *Gapdh *is broad with transcripts seen throughout the embryo except in the axial skeletal elements, where no expression is detected.

#### *Phosphoglycerate kinase 1, Pgk1* (MH2060, 1D, 5B)

*Pgk1 *is strongly expressed throughout the integuments, but *Pgk1 *transcripts are found in all organs with the exception of the axial skeleton (4D).

#### *Phosphoglycerate mutase 1, Pgam1* (MH1112, 4C, 5C)

Several tissues show strong *Pgam1 *expression including the ventricular zone, piriform cortex, cranial nerve nuclei, pituitary, olfactory epithelium, retina, dorsal root ganglia, cranial ganglia, whisker follicles, thymus, salivary glands, kidney and liver.

#### *Phosphoglycerate mutase 2, Pgam2* (MH2059, 3B)

The strongest *Pgam2 *expression is seen in the integuments, but expression is ubiquitous with the exception of the prevertebrae (4B).

#### *Enolase 1, alpha non-neuron enolase 1, Eno1* (MH2056, 3B, 4B)

*Eno1 *is expressed strongly in the integuments. With the exception of the prevertebrae, all tissues express *Eno1 *weakly.

#### *Enolase 2, gamma neuronal enolase2, Eno2* (MH1206)

*Eno2 *is characterized by strong expression in the CNS, cranial and dorsal root ganglia. *Eno2 *expression in the CNS, however, is highly regionalized with positive and negative tissues intermingling. For example, there is high expression in the piriform cortex but none in the surrounding cortical cells (1C). In the neocortex, *Eno2 *transcripts are detected in the marginal zone but not elsewhere (5A). In the pons, transcripts are abundant in the nuclei of the motor trigeminal nucleus (5D), but the rest of the pons shows much less expression. In the spinal cord, *Eno2 *expression is strongest in ventral-most neurons while very little signal is seen in the roof (5A). Outside the nervous system, expression of *Eno2 *is seen in salivary glands, and weakly in intestine and lung (5D).

#### *Enolase 3, beta muscle enolase 3, Eno3* (MH1229, 4A)

*Eno3 *is ubiquitously expressed except in the axial skeleton (3D).

#### *Pyruvate kinase, muscle, Pkm2* (MH1052, 2D, 4A)

Expression of *Pkm2 *is ubiquitous in the CNS but is elevated in certain structures such as the ventricular zone that lines the left ventricles and the inferior colliculus of the midbrain. Many tissues outside the CNS express this gene with the exception of the prevertebrae. In the liver, most cells do not express *Pkm2 *with the exception of blood vessels.

#### *Pyruvate kinase liver and red blood cell, Pklr* (MH1218, 3A)

*Pklr *mRNA is abundant in liver, lung, salivary gland, teeth primordia and gut (2C). Transcripts of this gene are also readily detected in the motor column of the spinal cord, in the olfactory epithelium and in intervertebral discs. Numerous other sites of expression are observed, but the signal strength is significantly lower than in the aforementioned tissues. *Pkm2 *and *Pklr *superimposed constitute a SEMP.

### Expression patterns of tricarboxylic acid cycle enzymes

Some of the 20 polypeptide chains catalyzing the 8 TCA cycle steps form multienzyme complexes (see Fig. 3B). This adds a new dimension to the analysis of expression because the components of multisubunit proteins should be coexpressed. Consequently, they are expected to exhibit similar expression patterns, which should be readily apparent in an ISH analysis. As discussed above, 12 of the 20 TCA cycle genes may also exhibit a SEMP. Although the variability within the SEMP is greater in the TCA cycle genes than in glycolysis, the stereotypic pattern is still observed. Using the same criteria as for glycolysis, matrix B in Fig. 3 provides a synopsis of expression and highlights SEMP results in bold. For each step of the TCA cycle, at least one gene exhibits a SEMP.

#### *Citrate synthase, Cs* (MH1408, 3A, 5A)

This gene is most strongly expressed in whisker follicles, cranial ganglia, dorsal root ganglia, salivary gland, differentiating neurons of the olfactory bulb and a subset of cranial nerve nuclei. Expression is also detected in liver, lung, kidney, heart and thymus.

#### *Citrate synthase like, Csl* (MH953, 3A, 5A)

This gene is broadly expressed throughout the embryo but signal strength is elevated in the cranial ganglia, liver, muscles, kidney, medulla, pons, heart, salivary gland, dorsal root ganglia, olfactory bulb, thymus, spinal cord, intestine, neocortex, midbrain and diencephalon. *Csl *is not expressed in the axial skeleton or the long bones of the limbs.

#### *Aconitase 1, Aco1* (MH1441, 3B, 5B)

The *Aco1 *gene is broadly expressed but stronger expression is seen in liver, kidney, lung, adrenal gland and salivary gland. Within the nervous systems, *Aco1 *transcripts are elevated in forebrain, brain stem, spinal cord, cranial and dorsal root ganglia.

#### *Aconitase 2, Aco2 *(MH1404, 3A, 4A)

Like *Aco1*, *Aco2 *is expressed in nearly all tissues. However, several sites are characterized by regional expression. For example, elevated expression is seen in the marginal zone of the neocortex (5C); in the hindbrain, localized expression is seen in several pontine (5B) and medullary nuclei (5B), and in the spinal cord, transcript levels are highest in the motor columns (4A). Dorsal root ganglia are characterized by intermingling of *Aco2-*positive and negative neurons (5A).

#### Isocitrate dehydrogenases complex

The conversion of isocitrate to alpha-ketoglutarate represents an allosterically regulated, rate-limiting step of the Krebs cycle. Five genes encoding isocitrate dehydrogenases have been identified in mammals (*Idh1*, *2*, *3a*, *3b *and *3g*). *Idh1 *(*Idpc*) encodes a cytosolic enzyme that uses NADP+ as cofactor and thus is not a Krebs cycle enzyme proper but has a major role in lipid metabolism [28]. *Idh2 *(*Idpm*) also encodes an enzyme that uses NADP+ as cofactor and is located in the mitochondrial matrix. *Idh3a*, *Idh3b *and *Idh3g *encode enzymes that utilize NAD+ as cofactor and reside in the mitochondrial matrix. These three polypeptides form a heterotetramer comprising two Idh3α subunits, one Idh3β and one Idh3γ subunit [29]. Idh3α has been proposed as the catalytic subunit while Idh3β and Idh3γ have supporting and regulatory roles [30]. It would be thus expected that these genes have similar expression patterns. Studies with recombinant polypeptides also suggest that isocitrate dehydrogenase heteromers are catalytically active only if they contain the *Idh3a *subunit [30].

#### *Isocitrate dehydrogenase 1, Idh1* (MH964, 3A, 4C)

*Idh1 *encodes a cytosolic enzyme that is strongly expressed in kidney, adrenal gland, intestine, thymus and cartilage. This gene is weakly expressed in stomach, facial soft tissue, muscle, lung and liver. *Idh1 *stands out because of its strong expression in CNS and PNS. It should be noted that Idh1 synthesizes alpha-ketoglutarate and NADPH, which can be utilized by glutamate synthase to generate the neurotransmitter glutamate, perhaps explaining the strong expression in the developing nervous system.

#### *Isocitrate dehydrogenase 2, Idh2* (MH1299, 1B, 3D)

Mitochondrial enzyme-encoding *Idh2 *is expressed nearly ubiquitously but the strongest signal is seen in skeletal muscles, the developing heart, choroid plexi (3D) and lens (1B). There is a notable absence of *Idh2 *expression in the developing axial and appendicular cartilage (1B).

#### *Isocitrate dehydrogenase 3 (NAD+) alpha, Idh3a* (MH965, 4C, 6A)

*Idh3a *is broadly expressed in the CNS with transcripts being observed in nearly all tissues. Nonetheless, this gene stands out among the isocitrate dehydrogenases because of its strong expression in muscles, heart, thymus, intestinal epithelium, liver, CNS, PNS, and developing whisker follicles. There is a notable absence of expression in the developing axial and appendicular cartilage, as is typical for SEMP.

#### *Isocitrate dehydrogenase 3 (NAD+) beta, Idh3b* (MH1313, 2A, 4A)

#### *Isocitrate dehydrogenase 3 (NAD+) gamma, Idh3g* (MH1300, 1D, 4A)

The expression patterns of *Idh3b *and *Idh3g *resemble that of *Idh3a*, although the intensity is much weaker. Thus, while *Idh3a *exhibits a definitive SEMP, this pattern is less pronounced for the β and γ subunits. The elevated expression of the α subunit can presumably be rationalized if we consider the stoichiometric ratios 2α:1β:1γ in the active enzyme and the implication of the α subunit as the catalytic enzyme, which could potentially drive the complex.

#### Oxoglutarate dehydrogenase complex

Oxoglutarate dehydrogenase is a mitochondrial enzyme complex comprising three different subunits (Ogdh, Dld, Dlst) that converts 2-oxoglutatate into succinyl-CoA and carbon dioxide. Like pyruvate dehydrogenase, oxoglutarate dehydrogenase forms a large multienzyme complex and the conversion of 2-oxoglutatate into succinyl-CoA requires the presence of all three subunits. Consequently, one would predict that *Ogdh*, *Dld *and *Dlst *genes have similar expression patterns.

#### *Oxoglutarate dehydrogenase lipoamide, Ogdh* (MH1551, 3D)

#### *Dihydrolipoamide S-succinyltransferase, Dlst* (MH948, 4A)

#### *Dihydrolipoamide dehydrogenase, Dld* (MH962, 4A)

All three genes encoding oxoglutarate dehydrogenase subunits show very similar expression patterns, but only *Ogdh *has strong SEMP features.

#### Succinate-coenzyme A ligase complex

Succinate-coenzyme A ligase is a mitochondrial matrix enzyme that converts succinyl-CoA to succinate and CoA. It consists of an invariant alpha subunit (encoded by *Suclg1*) and a beta subunit that is either ADP (*Sucla2*) or GDP-specific (*Suclg2*).

#### *Succinate-CoA ligase, alpha subunit, Suclg1* (MH1312, 4A)

#### *Succinate-coenzyme A ligase, ADP-forming beta subunit, Sucla2* (MH1492, 3A, 4A)

Both subunits exhibit the typical energy metabolism expression pattern with expression in the CNS, PNS, muscles, liver, kidney, gastrointestinal tract, adrenal and salivary glands. There is no expression in axial or limb cartilage. This stereotypical pattern is somewhat more pronounced for the ADP-forming beta subunit than for the alpha subunit.

#### *Succinate-Coenzyme A ligase, GDP-forming beta subunit, Suclg2* (MH2300)

Although *Suclg2 *expression resembles that of *Suclg *and *Sucla2 *in some ways, there are striking differences. Generally, CNS and PNS show very low *Suclg2 *expression. The only exceptions are in the ventricular zone lining the lateral ventricles of the forebrain, the lamina terminalis, the anterior boundary of the thalamus (4C) and the neuronal component of the pituitary (4B), where somewhat elevated expression can be seen. Other notable exceptions to the low expression of *Suclg2 *in the CNS are the scattered cells of the amygdala, which express the gene strongly (6D).

#### Succinate dehydrogenase complex

This complex consists of four subunits and is located in the inner mitochondrial membrane. Subunit A (Sdha) is a flavoprotein while subunit B is an iron-sulfur subunit (Sdhb). Subunits C and D are integral membrane proteins (Sdhc, Sdhd).

#### *Succinate dehydrogenase complex, subunit A, flavoprotein, Sdha *(MH2020, 4A, 5A)

#### *Succinate dehydrogenase complex, subunit B, iron sulfur, Sdhb* (MH1116, 3B, 4B)

#### *Succinate dehydrogenase complex, subunit C, integral membrane protein, Sdhc* (MH1427, 3A, 3D)

#### *Succinate dehydrogenase complex, subunit D, integral membrane protein, Sdhd *(MH1759, 4A)

Since all four polypeptide chains need to be coexpressed to form an active enzyme, it is not unexpected that the expression patterns of the four genes are related. There are, however, quantitative differences in expression level. For example, *Sdhd *expression in liver is much more pronounced than in other tissues (MH1759, 1B), while the expression of e.g. *Sdhb *is fairly similar everywhere (MH1116, 1B). Irrespective of such subtle quantitative differences, all four genes display the stereotypical energy metabolic enzyme pattern of expression.

#### *Fumarate hydratase 1, Fh1* (MH954, 2D, 4B)

This gene is broadly expressed and its pattern of expression resembles those of other glycolytic/TCA enzymes, although the differences in expression levels are less pronounced than those seen for *Idh3a *(Fig. 2).

#### Malate dehydrogenases

#### *Malate dehydrogenase 1, NAD+ (cytosolic), Mdh1* (MH1305)

Mdh1 is essential for the malate-aspartate shuttle, but like Idh1 it is not a component of the TCA cycle.*Mdh1 *is expressed in a SEMP-like manner with additional expression in the facial epithelia (2D).

#### *Malate dehydrogenase 2, NAD+ (mitochondrial), Mdh2* (MH1434, 3A, 4B)

*Mdh2*, a TCA cycle component, shows a SEMP expression pattern. The similarity in expression between *Mdh1 *and *Mdh2 *is critical since these enzymes play a pivotal role in the malate-aspartate shuttle. Since oxaloacetate cannot enter the mitochondrion directly, it is first converted by Mdh1 to malate, which readily enters the mitochondrion and is then reconverted to oxaloactetate by Mdh2.

Our study covers the expression of glycolysis and TCA cycle genes in full. At E14.5, at least one isoform of each step in these pathways shows a SEMP pattern. An exception to this rule is *Aldoa*, *Aldob *and *Aldoc*, none of which has a SEMP alone, though together they constitute a SEMP. A finding that emerges from our analysis of TCA cycle components is that in cases where the biochemistry depends on a multisubunit complex, the transcripts of at least one subunit show a SEMP while the other subunits may have uniform expression (Fig. 3B).

Some transcripts of glycolytic pathway genes are expressed highly regionally, such as *hexokinase isoform 4 *(*Gck*). This indicates a regional function more specialized than energy production. Northern blot data and ISH analysis of postnatal and adult tissues detect *Gck *mRNA in the lateral hypothalamic area and in the arcuate, ventromedial, dorsomedial and paraventricular nuclei [31,32]. It has been suggested that *Gck*-positive neurons are "glucose-sensing neurons" that respond to high glucose concentration [32]. The expression pattern of *Gck *at E14.5 suggests that a glucose-sensing mechanism has become established by this stage in development, since our data demonstrate *Gck *mRNA in the precursors of the above-mentioned hypothalamic nuclei.

Miki *et al*. [33] analyzed expression patterns in 49 different mouse embryonic and adult tissues using microarrays containing 18,816 mouse cDNAs. These authors found that genes encoding enzymes catalyzing glycolytic reactions that are closely linked have similar expression patterns. They also found that transcripts from key regulatory genes exhibited tissue specificity. To a large extent our data point in the same direction, although the data are obviously different in nature.

### Carbonic anhydrases

In mouse, 16 CA family members have been identified, 13 of which are CAs proper (see Background). Fifteen of the 16 members of this gene family were analyzed for expression at E14.5. The 15 RNA probes hybridized ranged between 900 and 1,600 nucleotides in length. For most of the CAs we were able to design templates, and hence probes, that had no sequence homology to any other family member. However, there was homology of ~ 80% in a few cases, ranging from 69 to 117 nucleotides (see Table 3). The highest homology over the longest stretch of DNA was seen between the *Car2 *probe and *Car3 *mRNA (77% over 168nts; Table 3). Despite this homology, no cross-hybridization occurred. For example, *Car2 *was strongly expressed in blood vessels of the brain and in the lung (MH1443), but no such expression was seen in these tissues in sections hybridized with authentic *Car3 *riboprobe (MH1651).

**Table 3:** Sequence homology matrix comparing carbonic anhydrase riboprobes and mRNA sequences

	**Carbonic anhydrase cDNA**							
	
**Template**	*Car1 *NM_009799^#^	*Car2 *NM_009801	*Car3 *NM_007606	*Car5a *NM_007608	*Car5b *NM_019513	*Car10-I *ENSMUST 00000092780*	*Car10II *ENSMUST 00000042943	*Car13 *NM_024495
*Car1*		77/98 (78%)§	78/96 (81%)					80/102 (78%)
*Car2*	77/98 (78%)		130/168 (77%)		84/112 (75%)			133/185 (71%)
*Car5b*		84/112 (75%)		117/149 (78%) 207/284 (72%)				
*Car5a*					117/149 (78%) 194/266 (72%)			
*Car7*				69/85 (81%)				
*Car11*						82/106 (77%)	82/106 (77%)	
*Car13*		80/102 (78%)						

# GenBank accession number of reference sequence

* Ensembl transcript ID

§Homology of aligned fragments using bl2seq (template/cDNA) and percent homology

### Expression patterns of carbonic anhydrases

#### *Carbonic anhydrase 1, Car1* (MH1800)

Strongly expressing cells are scattered throughout the liver (1A) and weak expression approaching background level is seen in various embryonic tissues.

#### *Carbonic anhydrase 2, Car2* (MH1443)

*Car2 *expression is regional. Major sites of expression are lung mesenchyme surrounding the bronchi, liver, capillaries distributed throughout the embryo, choroid plexi and cochlea (5B). In contrast to *Car1*, expression of *Car2 *is widespread.

#### *Carbonic anhydrase 3, Car3* (MH1651)

*Car3*, which encodes a cytosolic isozyme, is expressed with a high degree of tissue specificity. Striated muscles, facial cartilage (2B), ureter (3B) and scattered neurons in dorsal root ganglia express *Car3 *strongly. In liver, *Car3 *expression is widespread, resembling that of *Car2*.

#### *Carbonic anhydrase 4, Car4* (MH1444)

Expression of this gene, which encodes a protein anchored to the plasma membrane, is confined to developing glomeruli (5A) and weakly-expressing cells scattered throughout the CNS, presumably representing blood vessels. *Car4 *may be an excellent marker for embryonic kidney.

#### *Carbonic anhydrase 5a, mitochondrial, Car5a* (MH2294)

#### *Carbonic anhydrase 5b, mitochondria, Car5b* (MH1283)

No expression of these two mitochondrial enzymes is detected in the mouse embryo at E14.5.

#### *Carbonic anhydrase 6, Car6* (MH2295)

No expression is detected for the *Car6 *gene in the E14.5 mouse embryo.

#### *Carbonic anhydrase 7, Car7* (MH2296)

This cytoplasmic enzyme shows weak to medium expression throughout the embryo.

#### *Carbonic anhydrase 8, Car8* (MH2175)

Expression of this CA-RP is highly regional. Transcripts are abundant in cerebellum (2D), thyroid (4D), clavicle primordium, intestine and stomach (2D). *Car8 *is also detected in heart, dorsal aorta (4C), bronchi (5A), salivary glands and the cortical region of the kidneys (3C).

#### *Carbonic anhydrase 10, Car10* (MH2174)

Weak to medium expression can be seen scattered throughout the embryo, although there are also *Car10*-negative tissues such as the axial skeleton (3D). Somewhat elevated expression can be seen in scattered neurons of the CNS (3D) and PNS (2D) and in the heart (4D).

#### *Carbonic anhydrase 11, Car11* (MH2291)

*Car11 *is expressed in the cranial and dorsal root ganglia, olfactory bulb, striatum, amygdala and the midbrain. Particularly striking is the enrichment of these CA-RP transcripts in the boundary cap of the cranial (5D) and dorsal root ganglia (5C).

#### *Carbonic anhydrase 12, Car12* (MH2120)

This is another example of a regionally-expressed CA gene. Choroid plexi, salivary glands and facial as well as ventral integument, including the whisker follicles, express this transmembrane protein strongly (2D). A medium level of expression is detected in kidney, lung, cerebral cortex, hindbrain, spinal cord and the PNS.

#### *Carbonic anhydrase 13, Car13* (MH2292)

*Car13 *is strongly expressed in the inner ear and cochlea (3A). However, the most striking expression site of this gene is at a muscle fiber bundle located posterior to the submandibular gland (2D). Meninges covering the ventral delineation of the hypothalamus and pons exhibit attenuated expression (2C).

#### *Carbonic anhydrase 14, Car14* (MH1292)

This gene is expressed exclusively in the pigment layer of the retina (1A) and choroid plexi (3C).

#### *Carbonic anhydrase 15, Car15* (MH2293)

Strong expression is localized in cells scattered throughout the dorsal root ganglia (4D), dorsal spinal cord, pons, tegmentum and inferior colliculus (3D). A few other structures (bronchi, cornea and lens, for example) also express the gene, but only weakly.

Because carbonic anhydrases catalyze a ubiquitous reaction, one might have predicted them to be widely expressed. However, this was not the case, since half the CA genes showed a considerable degree of regionalization. Although there have been few reports about expression in the developing mouse embryo, we found our results to be generally consistent with published CA data. At comparable embryonic stages, transcripts of *Car3 *were reported in skeletal muscle and ureter [34], and *Car8 *mRNA was seen in cerebellum, gut, kidney, lung and heart [35,36]. The expression of Car12 in choroid plexus and kidney was also consistent with previous reports [37]. The specific expression sites reported here for *Car10*, *Car11*, *Car13 *and *Car15 *had not been described previously, although expression had been reported in whole embryos and in adult tissues using Northern blots and RT-PCR [9,11,36,38]. MA data for *Car1*, *14 *and *15 *showed them to be absent, but we were able to reveal definite and regionalized expression of these genes using ISH. MA analysis was apparently not sufficiently sensitive to detect mRNAs that are confined to a few cells representing a minute fraction of the total tissue from which the RNA was extracted.

The developmental expression we report here is also consistent with the expression described in adult tissues. Expression of embryonic *Car1 *[39], *4 *[40], *10*, *11 *[36], *12 *[38] and *14 *[41] presumably presages that in the adult. For example, adult Car 1 protein is abundant in erythrocytes [5,39]. The expression we describe for *Car1 *in the embryonic liver presumably marks sites of hematopoiesis because *Car1 *expression resembles that of hematopoietic maturation-associated *c-myb *(MH766) and the hepatocyte differentiation molecular marker *Hnf4α *(MH521) [42,43]. Furthermore, *Car1 *was recently suggested as a target of the transcription factor c-Myb [44]. Embryonic expression of *Car3 *in muscles and *Car4 *in glomeruli and blood vessels of the CNS presumably presages adult expression in these tissues [18,40]. In adult rat, Car4 has been proposed as an immunohistochemical marker for the blood-brain barrier and is thought to play an important role in CO_2_/HCO_3_^- ^homeostasis in the brain [40]. Expression in blood vessels of the CNS is already apparent in the E14.5 embryo, suggesting that CO_2_/HCO_3 _^- ^conversion is established early in development. Car5 has been reported as responsible for supplying HCO_3 _^- ^in gluconeogenesis and ureagenesis, pathways restricted to liver mitochondria [45,46]. Consistent with this, Car5 protein is only detected in adult liver [47]. Despite the substantial activities of all gluconeogenesis enzymes, gluconeogenesis itself is reported to proceed at very low rates in fetal hepatocytes and to be triggered by birth itself [48]. This is consistent with our inability to detect *Car5 *in the embryo. Finally, Car6 is normally secreted into saliva and milk [17,49]; there is no embryonic expression of *Car6*, perhaps because these fluids are not yet produced at E14.5, although salivary and mammary gland primordia are present at this stage of development.

## Conclusion

The results presented here constitute the first comprehensive description of the expression of all enzymes involved in glycolysis and the TCA cycle. Global studies such as this are especially useful for revealing higher degree-associations. We provide evidence for a Stereotypical Energy Metabolism Pattern (SEMP) of expression (Fig. 3). Of the total of 42 genes encoding either a glycolysis or a TCA cycle enzyme, 21 exhibited a SEMP. The defining future of a SEMP is expression in 22 "signature tissue". For the majority of genes classified as SEMP, expression in all of the 22 signature tissue is observed. Examples of exceptions are *Fh1*, *Sucla *and *Hk1*. The existence of a SEMP raises the possibility that many glycolytic and TCA cycle genes may be regulated in a coordinated fashion. Whether this is also true for other metabolic pathways, such as amino acid or protein syntheses, remains to be seen. Our study also makes the point that even so-called housekeeping genes, although quite broadly expressed, may vary significantly among tissues in levels of expression.

Kinases have received much attention during recent decades owing to their physiological importance and their high potential as drug targets. Initial efforts in the study of the kinome have concentrated on three specific aspects: *(i*) the identification of novel kinases, *(ii*) the identification of ligands to known kinases, and *(iii*) the complete characterization of signal transduction to map the biochemical pathways of the kinome fully [23,50]. As more data become available, so systematic and interactive databases arise, similar to that presented here for managing gene expression data [51,52]. Thus, our study complements the information gathered by the kinome-research community. At the same time, current trends in kinome research are switching towards structure prediction, structure analysis (to identify key amino acid residues) and gene expression profiling for eventual comparison in mutant or disease conditions [51,52]. The expression analysis of 190 kinases reported here sets the ground and illustrates the feasibility of a high-throughput expression analysis of the kinome, and provides the expression patterns for over one third of the mouse kinome.

With this study we also corroborate the practicality of high-throughput ISH. The usefulness of comprehensive data sets is even more apparent when all results are accessible through the Genepaint database [4]. Contrary to the common expectation that enzymes encoded by "housekeeping" genes will be widely expressed, almost half the carbonic anhydrase gene family shows a regionalized expression pattern and a number of them are so highly regionalized that they are suitable as tissue-specific markers. Hence these enzymes show regional diversity in expression, as reported for transcription factors [53] and growth factors [19], both of which are recognized key components of biological regulation.

In summary, this study greatly increases our general knowledge of the expression patterns of many genes that encode enzymes catalyzing various types of metabolic reactions. Although the data are from E14.5 embryos we believe that information collected at this stage serves as a suitable predictor and starting point for the elucidation of expression patterns in adult mice and presumably also in humans.

## Methods

### DNA template production

See Yaylaoglu et al. [19] for procedures.

### RNA probe synthesis

Probe synthesis was carried out as previously described [19]. The quantity and quality of riboprobes were assessed using the Bioanalyzer (Agilent Technologies, Waldbronn, Germany).

### Tissue collection, sectioning and in situ hybridization

All animal experiments were conducted in compliance with the German Law on Animal Welfare. Procedures for tissue collection, sectioning and ISH followed those described in Yaylaoglu et al. [19].

### Imaging and data management

Standard protocols as in Yaylaoglu et al[19] were followed. Images were acquired with a scanning microscope equipped with a 10× objective (NA 0.4) and a CCD camera programmed to capture 125 magnified frames per embryo section. Individual images were used to compose a mosaic image. Image resolution: 1.6 μm/pixel. Images were converted to Kodak FlashPix, which together with corresponding metadata were uploaded on to the Genepaint database [4].

## Abbreviations

CA Carbonic anhydrase

CA-RP CA-related polypeptides

ISH *in situ *hybridization

HT-ISH High-throughput *in situ *hybridization

MA Microarray

SEMP Stereotypical energy metabolism pattern

TCA Tricarboxylic acid cycle

CNS Central nervous system

PNS Peripheral nervous system

## Authors' contributions

Project concept and design: GE (group leader), OIK (group leader); template selection, primer design, DNA template production, RNA probe production: MC, AMH, Mehmet Ciftci, SB, HO, HB, IG, VC, TE, DE, MK, QJ; gene expression data acquisition and annotations: MC, AMH; manuscript drafting and critical reviewing: GE (group leader), OIK (group leader), AMH, MC. All authors read and approved the final manuscript.

## Supplementary Material

Additional File 1 **ISH expression pattern and microarray raw data (GSE6081) in the E14.5 mouse embryo**. This is a list containing all 662 genes analyzed in this study. Additional file 1 provides information regarding the expression of each gene integrated over the whole embryo. Expression was assessed by pattern (R, regional; U, ubiquitous; UWP, ubiquitous with pattern; N, not detected) and a numerical score for expression level (1, 2, 3). In all cases expression was assessed by one annotator and had to be confirmed independently by a second annotator. In (rare) cases of discrepancies, a consensus decision was used. Click here for file
